# Informational role self-efficacy: a validation in interprofessional collaboration contexts involving healthcare service and project teams

**DOI:** 10.1186/s12913-016-1382-x

**Published:** 2016-04-27

**Authors:** François Chiocchio, Paule Lebel, Jean-Nicolas Dubé

**Affiliations:** Telfer School of Management, Institut de recherche de l’Hôpital Montfort, University of Ottawa, 55 Laurier Avenue East, Ottawa, ON K1N 6N5 Canada; Department of Family Medicine and Emergency Medicine, Université de Montréal, Montréal, Canada; Internal Medicine Division and Critical Care Division Centre intégré universitaire de santé et de services sociaux de la Mauricie-et-du-Centre-du-Québec, Trois-Rivières, Canada

**Keywords:** Self-efficacy, Expertise, Interprofessional collaboration, Healthcare, Team

## Abstract

**Background:**

Healthcare professionals perform knowledge-intensive work in very specialized disciplines. Across the professional divide, collaboration becomes increasingly difficult. For effective teamwork and collaboration to occur, it is considered necessary for individuals to believe in their ability to draw on their expertise and provide what others need to perform their job well. To date, however, no instruments exist to measure such a construct.

**Methods:**

A two-study design is used to test the psychometric properties, factor structure and incremental validity of a five-item questionnaire measuring informational role self-efficacy.

**Results:**

Based on parallel analysis and exploratory factor analysis, Study 1 shows a robust and reliable one-dimensional construct. Study 2 cross-validates this factor structure using confirmatory factor analysis. Study 2 also shows that informational role self-efficacy predicts proactive teamwork behaviors over and above goal similarity, interdependence, coordination and intra-team trust.

**Conclusions:**

The instrument can be used in research to assess an individual’s capability beliefs in communicating his/her informational characteristics that are pertinent to the task performance of others. The construct is also shown to have value in team-building exercises.

**Electronic supplementary material:**

The online version of this article (doi:10.1186/s12913-016-1382-x) contains supplementary material, which is available to authorized users.

## Background

### Complexity and informational silos in healthcare

The expanding rate of new knowledge in technical fields fosters the need for greater specialization and the development of subfields [1]. This phenomenon is echoed in healthcare [2] where healthcare professionals perform complex knowledge-intensive tasks [3]. The more healthcare professionals must train to integrate knowledge vertically and grasp the complexity of their specific profession, discipline or field—digging deeper as they specialize—the more challenging it is for them to integrate knowledge horizontally to collaborate interprofessionally. Hood [4] describes this problem in terms of a “double reflexivity”; a “double hermeneutic”. Unfortunately, in healthcare “members of each profession know very little of the practices, expertise, responsibilities, skills, values and theoretical perspectives of professionals in other disciplines” [5] in spite of the fact that communicating about expertise fosters trust [6]. Information about one’s training background, expertise and knowledge is in itself a type of knowledge referred to as *informational characteristics*: the “underlying attributes of individuals (e.g., work experience and education) which, although not immediately detectable, are important in the completion of the task” [7].

One key in helping healthcare professionals to better share informational characteristics and work collaboratively is to examine the core beliefs anchoring their actions. Many studies have shown that if a person believes he/she can perform a specific task well, he/she usually does [8] because the belief is accompanied by additional efforts and persistence when facing difficulties [9]. Such beliefs are called self-efficacy. Self-efficacy is an individual-level construct defined as beliefs in one’s capabilities to succeed at something specific [9, 10]. Meta-analyses on the topic show moderate to high corrected correlations in various work settings [11–13]. A specific example in healthcare shows a positive relationship between increases in social service workers’ creative self-efficacy and increases in creative problem-solving and innovation [14].

Despite the central role of knowledge integration in interprofessional collaboration there are—to our knowledge—no instruments that measure the underlying beliefs of individuals in sharing their expertise. Achieving this through the development of a robust, specific, short, and one-dimensional measure is therefore the goal of the present study.

### Incremental validity over teamwork “building blocks”

When a new measure is introduced, a stringent verification of its utility is to test for incremental validity; that is, to ask whether the measure adds to the prediction of a criterion above what can be predicted by existing phenomena and measures [15]. Four such criteria are crucial in predicting individual behaviors of teamwork and collaboration: goal similarity, interdependence, coordination, and trust.

A team is a collection of individuals united by a common goal and task interdependence [16, 17]. “Shared goals are what make collaboration ‘collaborative.’ Without at least one shared goal or endpoint, there would be no reason for two or more entities to work together at all” [18]. Team effectiveness hinges on dynamic and adaptive management of interdependencies [19]. Clarifying interdependences between team mates with different roles, and explicitly ironing out work processes through task-oriented coordination lead to team effectiveness [20]. Trust is another construct a new measure should add value to. Trust is crucial in high autonomy teams engaged in ambiguous, unstructured and novel situations [21]. An emergent state resulting from social interactions over time [22], trust derives from behaviour reliability (i.e., calculus-based trust) or from shared values and ideas (i.e., identification-based trust) [23]. Crucially, trust is also about people understanding each other—understanding without which individuals “will have trouble benefiting from the expertise of others” [24]. Attributions of trustworthiness (or lack thereof) can be based on a mix of previously held beliefs about another group (e.g., a professional group) and further thought processes prompted by new information about that group [25]. In healthcare settings particularly, trust is based on individuals manifesting competence including how competence is communicated [26]. Consequently, we posit that:

Informational role self-efficacy will demonstrate incremental validity over perceptions of goal similarity, interdependence, coordination, and intra-team trust, in predicting proactive team behaviors.

Two studies are needed to test this hypothesis, the first to develop the instrument and provide solid psychometric properties, and the second to cross-validate the instrument’s properties with a new sample and test for incremental validity.

## Method

### Study 1

#### Construct definition and instrument development

Based on Jehn, Bezrukova, and Thatcher’s definition of informational characteristics (e.g., work experience and education) [7], on Murphy and Jackson’s definition of a work role as “the total set of performance responsibilities associated with one’s employment” [27], on Conway’s definition of task performance as “job-specific behaviors including core job responsibilities, for which the primary antecedents are likely to be ability and experience” [28], and on self-efficacy’s focus on specific beliefs in one’s capabilities to produce given attainments [9], we define informational role self-efficacy as *an individual’s capability beliefs in communicating his/her informational characteristics that are pertinent to the task performance of others*.

In developing a preliminary version of the scale, we were led by three guiding principles relevant to scale construction in general and self-efficacy scale development in particular. These three guiding principles are practicality, conceptual footing, and technical quality.

In terms of practicality, scales need to be developed with a specific intent which then becomes the backdrop against which its validation is conducted and assessed [29]. As such, assessing behaviors provide concrete anchors useful for self-regulation and feedback. Low scores point to the need to build capability for specific behaviors. The first two authors conducted discussions with healthcare professionals and academics to bring forward examples of behavioral manifestations indicative of an individual’s contribution to taskwork and teamwork in terms of informational characteristics, with an emphasis on manifestations that are perceptible across professional and disciplinary boundaries.

The second guiding principle refers to the conceptual footing on which the scale is erected. In parallel to the inductive approach just described, and because development of the instrument must consider content validity [30], we adopted a deductive framework based on two sets of concepts. The first concept is that items should address the efficacy domain very specifically and reflect behaviors or actions that are under one’s control, following Bandura [31]. The second concept is teamwork and collaboration which represents the target context in which individuals are expected to function. Accordingly, the first two authors scanned the literature for relevant behavioral indicators and discussed corresponding examples.

The third and final guiding principle pertains to technical quality. Capability statements must be unambiguous (e.g., the survey must avoid double-barrel questions), have fewer than 15 words, contain only one verb and employ the active voice [30, 32]. The response scale must be positive only, allow for sufficient variability, and ask people to self-assess on what they can or cannot do [31].

With these inductive, deductive, and technical constraints in mind, the first two authors developed a response scale and wrote 11 capability statements.

#### Participants and procedures

Study 1 adhered to the Helsinki Declaration and was granted ethics approval CHUM-09.287 by the Centre hospitalier de l’Université de Montréal. Three hundred and eleven (311) critical care professionals from four intensive care units at the hospital signed a consent form and agreed to complete a paper-and-pencil questionnaire. In addition to basic demographic questions (i.e., age, sex), they answered the 11 professional role self-efficacy items.

We examined responses for missing values, univariate, and multivariate normality [33]. Among 3,421 answers collected (i.e., 311 participants X 11 items), 22 (0.64 %) were incomplete and were replaced by the mean. Inspection of each item’s distribution revealed normal skewness and kurtosis for five items. The other six items were removed from further analysis. Multivariate normality tests using Mahalanobis distance revealed 21 participants who exceeded the cut-off value of 20.515 for 5-item questionnaires; data from these participants was deleted.

The final sample consisted of 290 participants: 69 men (23.8 %) and 221 women (76.2 %) working together in three categories: physicians (*N* = 44, 15.2 %), nurses (*N* = 175, 60.3 %), and other critical care professionals (*N* = 71, 24.5 %). The proportion of men and women differs as a function of profession (*χ*^2^_(2)_ = 35.8; *p* < 0.005), with more women working as nurses and other professionals (e.g., pharmacists, respiratory therapists) than physicians (i.e., intensivists, fellows, residents). Overall, participants had a mean age of 36.5 (*SD* = 9.9).

### Study 2

#### Participants and procedures

Study 2 adhered to the Helsinki Declaration and was granted ethics approval CERFAS-2009-10-050-A by the Faculté des Arts et des Sciences de l’Université de Montréal. Data was collected as part of a larger research project which examined the efficiency and efficacy of interprofessional healthcare teams involved in a project [34]. The researchers contacted the human resource departments of several healthcare establishments in a large North-American city to identify teams comprised of at least five members representing at least three healthcare professions or disciplines. In order to participate, the teams could not be related to Study 1 and they had to be involved in a project. Participants working on 14 projects in nine establishments agreed to take part in the study. An example of a project was to implement fluid interprofessional evaluative processes for adult psychiatric patients requiring specialized care for cardio-metabolic pathologies. Each participant signed a consent form. In order to avoid common method variance issues [35] informational role self-efficacy was assessed before or in the early stage of the project and teams’ existence. All other variables were measured, on average, 18.4 weeks later (*SD* = 8.2).

Because we used electronic questionnaires, there were no missing data within each measurement time. All variables showed normal univariate distributions. Multivariate normality tests using Mahalanobis distance did not reveal multivariate outliers [33]. In spite of the fact that 109 persons participated at Time 1, 77 persons responded at the two measurement times: 22 men (28.6 %) and 55 women (71.4 %) working as physicians (*N* = 14, 18.2 %), nurses (*N* = 17, 22.1 %), other professionals (*N* = 42, 54.5 %), and support personnel (*N* = 4, 5.2 %). The proportion of men and women differed as a function of profession (*χ*^2^_(3)_ = 18.3; *p* < 0.005) with more men in the physician category. Other descriptive statistics and reliabilities are shown in Table 1.

**Table 1:** Descriptive statistics and correlations among Study 2 variables (*N* = 77)

	*M* (*SD*)	1	2	3	4	5	6	7	8	9
1. Age	42.33 (10.5)	---								
2. Sex	---	.068	---							
3. Profession	---	-.226*	-.146	---						
4. Informational role self-efficacy	79.4 (13.3)	.174	-.152	.142	.924					
5. Inter-dependence	3.72 (0.69)	.183	-.021	-.043	.228*	.689				
6. Goal similarity	3.90 (0.54)	.222	-.229*	-.071	.198	.440**	.785			
7. Explicit coordiation	3.26 (0.98)	.136	-.189	.084	.004	.261*	.458**	.914		
8. Intra-team trust	4.11 (0.62)	.076	-.158	-.003	.320**	.079	.336**	.243*	.890	
9. Proactive team performance.	3.27 (1.07)	.314*	.051	-.009	.388**	.236*	.402**	.418**	.406**	.928

Note

Sex: 1 = Women, 2 = Men; Profession 1 = Physicians, 2 = Nurses, 2 = Professionals, 4 = Support

Diagonal shows Cronbach's alphas

*p < 0.05; **p < 0.01

#### Measures

In addition to the five informational role self-efficacy items shown in the Additional file 1, study participants answered to additional scales. We measured task interdependence with Campion, Medsker, and Higgs’s [36] 3-question scale (e.g., *Within my project team, jobs performed by members are related to one another*). We measured goal similarity using Jehn’s [37] 3-item measure of this construct (e.g., *In my project team, we have similar goals*). Intra-team trust was measured using Simons and Peterson’s [38] 5-item measure (e.g., *We are all certain that we can fully trust each other*). These three instruments used a 5-point response format (i.e., 1 = strongly disagree; 5 = strongly agree). Coordination was measured with an instrument validated in ongoing service delivery and project contexts [39] (e.g., *In my team we discuss information on ‘who does what’*) using a frequency response format (i.e., 1 = never or almost never; 2 = occasionally. 3 = relatively often; 4 = often; 5 = very often). Individual-level performance behaviors relevant to teamwork are taken from Griffin et al. [40]. We used their 3-item proactivity scale (e.g., *I suggested ways to make our team more effective*) with the same frequency answer format as for coordination.

## Results

### Study 1

To assess the stability of the scale across gender and profession, we conducted six principal component and factor analyses: one with the total sample and five with subsamples of nurses, physicians, professionals, men, and women only. Because of the inappropriateness of using the “eigenvalue > 1 rule” to determine the number of factors present [41], we performed parallel analysis for all six principal component analyses using Hayton, Allen, and Scarpello’s [42] statistical routines. These analyses showed that only one factor is present overall and within each five sub-samples. We then proceed with principal axis factor analyses specifying a single factor. Results appear in Table 2. Together, these results show very strong support for a short single-factor construct measured reliably across profession and gender.

**Table 2:** Summary of Study 2’s principal axis factor analyses

	Complete sample (*N* = 290)	Nurses (*N* = 175)	Physicians (*N* = 44)	Profes. (*N* = 71)	Men (*N* = 69)	Women (*N* = 221)
Kaiser-Meyer-Olkin index	0.893	0.894	0.819	0.844	0.883	0.887
Barlett’s test of sphericity	1211.4*	705.7*	190.9*	273.6*	268.5*	884.7*
% of total variance explained	74.6	73.7	75.1	71.2	73.8	73.3
Cronbach’s alpha	0.936	0.933	0.934	0.924	0.933	0.932
*M*	76.84	73.66	83.25	80.70	83.15	74.87
*SD*	14.51	15.04	12.71	11.90	12.15	14.65

*p < .001

### Study 2

Given the single-factor structures seen across sub-samples in Study 1, a strong test of the measurement model and single-factor theoretical structure involves using confirmatory factor analysis to cross-validate Study 1’s results onto Study 2's sample. Confirmatory factor analysis shows the single-factor structure adjusts well to the data given the relatively small sample size (i.e., *χ*^2^_(5)_ = 6.23; *p* = 0.28; CFI = 0.983; NNF = 0.993; NNFI = 0.993; IFI = 0.996; SRMR = 0.02, and RMSEA = 0.05 with 90 % CI between 0.00 and 0.155). Table 3 shows incremental validity evidence; that is the extent to which informational role self-efficacy contributes to the prediction of dependent variables over control variables and teamwork “building blocks”. We can see that informational role self-efficacy is a positive predictor of intra-team trust (Beta = 0.308, *p* < 0.01) adding 7.3 % (*p* < 0.05) variance over and above what age, sex, profession, goal similarity, interdependence, coordination, and intra-team trust already contribute to the prediction of proactive performance. Our hypothesis is supported by the results.

**Table 3:** Hierarchical multiple regression predicting proactive team performance in Study 2 (*N* = 77)

	Standardized Beta		
	Model 1	Model 2	Model 3
Control variables			
Age	0.327**	0.209*	0.151
Sex	0.039	0.175	0.212*
Profession	0.070	0.033	−0.027
Teamwork building blocks			
Goal similarity		0.043	−0.027
Interdependence		0.152	0.152
Coordination		0.267*	0.327**
Intra-team trust		0.299**	0.201‡
Informational role self-efficacy			0.308**
* R* ^2^	0.104*	0.378**	0.451**
△*R* ^2^		0.274**	0.073*

Note

Sex: 1 = Women, 2 = Men; Profession 1 = Physicians, 2 = Nurses, 2 = Professionals, 4 = Support

‡ *p* = 0.053; *p < 0.05; **p < 0.01

## Discussion

### Implications for research

We see at least two streams for future direction in research: team type and power heterarchy. First, because ongoing service delivery and project work are different forms of work [43] healthcare professionals involved in one may not play the same roles when involved in the other [44]. In healthcare service work, individuals acquire professional expertise early on in their training and this is an important factor in the formation of professional boundaries. Because interprofessional collaboration is seen as a determinant of high quality patient care [45], one’s ability to share expertise across expertise-based knowledge silos is very important. However, because project work primarily centres on organisational issues (i.e., “fixing” the system) rather than directly focussed on patient care (i.e., “fixing” the patient), healthcare expertise is still necessary when working on a project but less so than when providing healthcare to patients. Accordingly, we expect that the impact of informational role self-efficacy will be stronger in regular ongoing healthcare service work compared to project work.

Second, power heterarchy within teams is “a relational system in which the relative power among team members shifts over time as the resources of specific team members become more relevant (and the resources of other members become less relevant) because of changes in the situation or task” [46]. Power dynamics among physicians and nurses is a well-documented inhibitor of interprofessional collaboration in ongoing healthcare service work [5]. Since information is a resource and a form of power [47], we suggest future studies should test whether minimal interprofessional collaboration occurs because of nurses’ thwarted informational role self-efficacy. If so, building capacity beliefs in nurses should improve their collaborative behaviors.

### Implications for practice

Organizations can shape employees’ sense of efficacy towards desired performance outcomes [14]. Organizations can also engage in team-level activities. Discussing the five behaviors of our scale in a team-building exercise is pertinent for two reasons. First, the five behaviors relate to roles and communication, which is significant in light of a recent meta-analysis showing that team-building has an important effect on individuals’ role clarification and communication [48]. Second, team members tend not to share what is unique to each other and prefer discussing what is common to the team [49]. Consequently, it is important to make a conscious effort to stimulate team discussions on the informational roles of individual team members because, by definition, these roles will vary according to differences in individual expertise. These two reasons concur to suggest that a group discussion followed by an action plan aiming at increasing the quantity and quality of the five behaviors of our scale are likely to have a positive impact on how individuals collaborate.

### Limitations and future research directions

This study has three limitations, each with implications for future research. First, the validity evidence is limited to a single method. Although measures are separated in time by 18 weeks on average, there is nevertheless a possible method effect [35]. Future studies should measure dependent variables with a variety of methods (e.g., questionnaires, observations, interviews). Second, our sample size for Study 2 is small which hinders generalizability. Third, informational role self-efficacy is an individual-level construct and our studies were not designed to draw conclusions at the team level. While we were careful to interpret results in terms of individual perceptions of team phenomena [50] and used instruments designed to assess individual behavior manifestation and/or perceptions pertinent to the team context [40], a worthy area of future research would be to integrate our measure in multi-level designs [51]. One such study could measure the relationship between (individual-level) informational role self-efficacy and task performance as a function of collective efficacy (group level). Collective efficacy is a shared belief that the team is able to perform effectively [52–54]. It is logical to hypothesize that the relationship between informational role self-efficacy and task performance will be stronger in teams that rate high in collective efficacy compared to teams with low collective efficacy. Despite these limitations, we demonstrated that informational role self-efficacy is a measurable single-factor individual-level construct and is a correlate and predictor of important phenomena necessary in today and tomorrow’s diversified and dynamic work environment.

## Conclusion

These studies are the first to address one’s predisposition and capability beliefs regarding communicating the expertise that others need to perform their job well with the introduction of an instrument that measures informational role self-efficacy. Cross-validation evidence using exploratory and confirmatory factor analyses supports a robust one-dimensional construct measured with a short 5-item behavioral self-assessment. Over an 18-week period, informational role self-efficacy adds variance to the prediction of perceptions proactivity behaviors over and above perceptions of shared goals, interdependence, coordination, and intra-team trust. These results suggest the instrument will have value in future theoretical and conceptual work as well as in practical contexts focusing on interprofessional collaboration.

## Availability of data and materials

Data is not available publicly because informed consent did not include publication of raw data.

## Additional file

Additional file 1: Informational role self-efficacy scale. (DOC 33 kb)
