# Supplementary material for: Informational role self-efficacy: a validation in interprofessional collaboration contexts involving healthcare service and project teams
Source: BMC Health Serv Res. 2016 Apr 27;16:153. doi: 10.1186/s12913-016-1382-x (PMC4847374; doi:10.1186/s12913-016-1382-x)
Supplement: Additional file 1: — Informational role self-efficacy scale. (DOC 33 kb) [file 12913_2016_1382_MOESM1_ESM.doc]

**Additional file 1**

**Informational role self-efficacy scale**

| We all have special training and knowledge that have the potential to contribute to teamwork. The following list of statements describes activities that relate to your expertise. Assess how confident you are in your ability to perform these activities by associating each activity with any number between 0 % and 100 % using the following scale: | | | | | | | | | | | | | |
| --- | --- | --- | --- | --- | --- | --- | --- | --- | --- | --- | --- | --- | --- |
|  | | | | | | | | | | | | | |
| 0 % | 10 % | | 20 % | 30 % | 40 % | 50 % | 60 % | 70 % | 80 % | | 90 % | | 100 % |
| I cannot do this activity | | | |  | I am moderately certain I can do this activity | | |  | I am entirely certain I can do this activity | | | | |
|  | | | |  |  | | |  |  | | | | |
| 1. | | Build on my area of ​​expertise to enrich team discussions | | | | | | | |  | | % | |
| 2. | | Improve teamwork by interventions that showcase my professional expertise | | | | | | | |  | | % | |
| 3. | | Clarify the nature of my professional expertise with team members | | | | | | | |  | | % | |
| 4. | | Advise team members by integrating the specifics of my area of expertise | | | | | | | |  | | % | |
| 5. | | Show the contribution of my area of expertise when the team needs to solve a problem | | | | | | | |  | | % | |
